# Effects of Tributyltin Chloride on Cybrids with or without an ATP Synthase Pathologic Mutation

**DOI:** 10.1289/EHP182

**Published:** 2016-04-29

**Authors:** Ester López-Gallardo, Laura Llobet, Sonia Emperador, Julio Montoya, Eduardo Ruiz-Pesini

**Affiliations:** 1 Departamento de Bioquímica, Biología Molecular y Celular,; 2 Instituto de Investigación Sanitaria de Aragón,; 3 CIBER de Enfermedades Raras (CIBERER), and; 4 Fundación ARAID, Universidad de Zaragoza, Zaragoza, Spain

## Abstract

**Background:**

The oxidative phosphorylation system (OXPHOS) includes nuclear chromosome (nDNA)– and mitochondrial DNA (mtDNA)–encoded polypeptides. Many rare OXPHOS disorders, such as striatal necrosis syndromes, are caused by genetic mutations. Despite important advances in sequencing procedures, causative mutations remain undetected in some patients. It is possible that etiologic factors, such as environmental toxins, are the cause of these cases. Indeed, the inhibition of a particular enzyme by a poison could imitate the biochemical effects of pathological mutations in that enzyme. Moreover, environmental factors can modify the penetrance or expressivity of pathological mutations.

**Objectives:**

We studied the interaction between mitochondrially encoded ATP synthase 6 (p.MT-ATP6) subunit and an environmental exposure that may contribute phenotypic differences between healthy individuals and patients suffering from striatal necrosis syndromes or other mitochondriopathies.

**Methods:**

We analyzed the effects of the ATP synthase inhibitor tributyltin chloride (TBTC), a widely distributed environmental factor that contaminates human food and water, on transmitochondrial cell lines with or without an ATP synthase mutation that causes striatal necrosis syndrome. Doses were selected based on TBTC concentrations previously reported in human whole blood samples.

**Results:**

TBTC modified the phenotypic effects caused by a pathological mtDNA mutation. Interestingly, wild-type cells treated with this xenobiotic showed similar bioenergetics when compared with the untreated mutated cells.

**Conclusions:**

In addition to the known genetic causes, our findings suggest that environmental exposure to TBTC might contribute to the etiology of striatal necrosis syndromes.

**Citation:**

López-Gallardo E, Llobet L, Emperador S, Montoya J, Ruiz-Pesini E. 2016. Effects of tributyltin chloride on cybrids with or without an ATP synthase pathologic mutation. Environ Health Perspect 124:1399–1405; http://dx.doi.org/10.1289/EHP182

## Introduction

The mitochondrial DNA (mtDNA) m.8993T>G transversion in the *MT-ATP6* gene provokes a p.L156R substitution in transmembrane helix 4 (TMH4) of the p.MT-ATP6 subunit. This polypeptide is an ATP synthase (complex V, CV) component of the oxidative phosphorylation system (OXPHOS). The amino acid position 156 is located in the channel used by protons to enter the mitochondrial matrix and power ATP synthesis. The m.8993T>G mutation is associated with maternally inherited Leigh syndrome (MILS) and neurogenic muscle weakness, ataxia, and retinitis pigmentosa (NARP) (Holt et al. 1990; Thorburn and Rahman 1993). In general, NARP is caused by moderate levels of the m.8993T>G mutation, whereas individuals with mutant loads > 90% have MILS (Tatuch et al. 1992). However, in some families, oligosymptomatic children share the same mutation load as those of symptomatic siblings (Enns et al. 2006), and high mutation loads are not always associated with signs of MILS or NARP (Degoul et al. 1995; Mkaouar-Rebai et al. 2009; Tsao et al. 2001). Similarly to many other pathological mutations (Cooper et al. 2013; Lake et al. 2015), other factors are likely involved in the phenotypic differences among individuals with the same m.8993T>G mutation load.

Other pathological mutations in mtDNA genes have been reported in MILS patients (Montoya et al. 2009; Ruhoy and Saneto 2014; Thorburn and Rahman 1993). Thus, polymorphic variation in these genes may influence the m.8993T>G phenotype. Indeed, the mtDNA genetic background (mtDNA haplogroups) plays an important role in modulating the biochemical defects and the clinical outcome by altering the risk of MILS caused by m.8993T>G (D’Aurelio et al. 2010; Hao et al. 2013). Moreover, many other pathological mutations in nuclear DNA (nDNA) genes have been described in Leigh syndrome (LS) patients (Ruhoy and Saneto 2014). For example, a mutated mitochondrial aminoacyl-transfer RNA (tRNA) synthetase was described in a patient with LS (Schwartzentruber et al. 2014). Interestingly, a patient with MILS had a homoplasmic mutation in the tRNA^Val^, and his clinically normal mother was also homoplasmic mutant (McFarland et al. 2002). It was recently shown that overexpression of mitochondrial valyl-tRNA synthetase can restore the steady-state levels of the mutated tRNA^Val^. Thus, interindividual variations of this synthetase may underlie clinical differences (Rorbach et al. 2008). Therefore, polymorphic variation in nDNA genes may influence the m.8993T>G phenotype.

In addition to nuclear and mitochondrial genetic factors, environmental stimuli may modify the phenotype of the m.8993T>G mutation. Certain chemicals trigger the appearance of pathological phenotypes associated with mtDNA mutations. For example, individuals harboring the m.1555A>G transition in the *MT-RNR1* gene for 12S ribosomal RNA (rRNA) suffer nonsyndromic hearing loss when exposed to aminoglycosides (Prezant et al. 1993). Furthermore, occupational exposure to *n*-hexane and other solvents precipitated visual failure in a Leber hereditary optic neuropathy patient with the m.11778G>A mutation (Carelli et al. 2007). Previous reports suggest that the CV proton channel, particularly the p.MT-ATP6 subunit, is the target site for organotin compounds, including tributyltin chloride (TBTC) (von Ballmoos et al. 2004). These compounds contaminate human food and water (Kotake 2012). Therefore, it is possible that TBTC affects the expressivity of the m.8993T>G mutation.

## Methods

### Transmitochondrial Cell Line Construction, Characterization and Functional Investigations

To homogenize nuclear and environmental factors, we built transmitochondrial cell lines (cytoplasmic hybrids or cybrids) with osteosarcoma 143B or adenocarcinoma A549 rho^0^ nuclear backgrounds using patient and control platelets (Chomyn et al. 1994). All samples were collected with written informed consent, and the Ethics Review Committees of the involved hospitals and the Government of Aragón approved the study [Comité Ético de Investigación Clínica de Aragón (CEICA) 11/2010].

The cybrids were grown in Dulbecco’s modified Eagle medium (DMEM) containing glucose (1 g/L), pyruvate (0.11 g/L) and fetal bovine serum (5%) with no antibiotics (Llobet et al. 2015a).

For molecular cytogenetic analysis, cells were exposed to colchicine (0.5 μg/mL) for 4 hr at 37°C and were harvested routinely. Metaphase cells were prepared following a conventional cytogenetic protocol for methanol:acetic acid (3:1)–fixed cells. Approximately 20 metaphase cells were captured and analyzed for each cell line. The genetic fingerprint of the cybrid cell lines was determined using an AmpFLSTR® Identifiler® PCR Amplification Kit (Life Technologies) and an ABI Prism 3730xl DNA analyzer (Applied Biosystems). These genetic fingerprints were compared with those from the American Type Culture Collection (ATCC) cell lines. To confirm the nucleotide at the m.8993 position, a polymerase chain reaction–restriction fragment length polymorphism (PCR-RFLP) analysis was performed (López-Gallardo et al. 2014). mtDNA sequences were obtained using a BigDye Terminator v.3.1 Cycle Sequencing Kit (Applera Rockville) and an ABI Prism 3730xl DNA analyzer. The revised Cambridge reference sequence (GenBank NC_012920) and an mtDNA phylogenetic tree were used to locate mutations and to define mtDNA haplogroups (van Oven and Kayser 2009), respectively. The level of peroxisome proliferator-activated receptor gamma (PPARγ) messenger RNA (mRNA) was determined in triplicate using reverse transcriptase–quantitative PCR (RT-qPCR) and the One-Step Real-Time system (Applied Biosytems). The expression levels were normalized using 18S rRNA. The ΔCt method was used to calculate fold expression. StepOne software v.2.0 (Applied Biosystems) was used for data analysis.

Analyses of oxygen consumption, ATP, and H_2_O_2_ levels were performed in triplicate according to previously described protocols (Gómez-Durán et al. 2010). Determination of the mitochondrial inner membrane potential (MIMP) was performed using a Mito-ID Membrane Potential Detection Kit (Enzo Life Sciences). Fluorescence microscopy was performed on live cells using a Floyd Cell imaging station (Life Technologies). Isocitrate dehydrogenase (IDH) activity was determined using a commercial Isocitrate Dehydrogenase Colorimetric Assay Kit (Abcam®) according to the manufacturer’s instructions. Briefly, 1 × 10^6^ cells grown in DMEM were lysed in 200 μL of an assay buffer provided in the kit. The lysate was centrifuged at 13,000 × *g* for 10 min, and the cleared supernatant was used for the assay. NAD^+^ was used as the substrate for the NAD-IDH assay. Measurements were obtained using a NovoStar MBG Labtech microplate instrument.

In a previous study, 25 of 32 blood donors from the state of Michigan, USA, showed detectable concentrations of TBT in whole blood samples (Kannan et al. 1999). The observed range, 8.3–293 nM, encompasses the TBTC concentrations used in the present study. When required, TBTC (Sigma-Aldrich) or oligomycin (OLI) (Sigma-Aldrich), another CV inhibitor, was dissolved in ethanol and added to the respiration medium during the oxygen consumption determination and to the medium for ATP, MIMP, H_2_O_2_, or IDH determination during the 2-hr, 15-min, 30-min, or 24-hr incubation periods, respectively.

### Statistical Analysis

The statistical package StatView 6.0 (SAS Institute Inc.) was used to perform all statistical analyses. The data are presented as the mean and standard deviation. At least three analyses were performed for each parameter. An unpaired two-tailed *t*-test was used to compare parameters. Linear regression analyses were performed, and linear regression equations and regression coefficients are indicated for TBTC concentrations versus oxygen consumption, ATP amount, or H_2_O_2_ level. *p*-Values < 0.05 were considered statistically significant.

## Results

### Characterization of the Cybrid Cell Lines

Five cybrid cell lines were built. Two cybrids within the adenocarcinoma A549 nuclear background were intended to harbor the m.8993T (Awt) or m.8993G (Am) mtDNA alleles. The other three cybrids within the osteosarcoma 143B nuclear background were also intended to harbor the m.8993G (Om) or m.8993T mtDNA alleles. For the osteosarcoma 143B m.8993T cells, a nonisogenic wild-type cybrid was first generated (Owt). An isogenic cybrid was produced (Owti) after we obtained platelets from the wild-type mother of the patient harboring the mutation.

Karyotyping was used to verify that the nuclear backgrounds were equivalent. Thus, the Am and Awt cybrids shared the modal number of chromosomes (60) and several chromosomal abnormalities [del(2p21), +der(6)t(1;6)(q24–25?;q23), +der(7)delq32?, del(11q23)] (see Figure S1). The modal number of chromosomes differed in Owt (66), Owti (69), and Om (70/71) cybrids; however, these numbers are similar to those previously published in other osteosarcoma 143B transmitochondrial cell lines (Gómez-Durán et al. 2010). These cybrids shared several chromosomal abnormalities [+i(7p), +der(7)t(1;7)(q25;q32)[2], +der(12)add(q24.3)]. To confirm the celluar origin of our cybrids and the equivalence of their nuclear backgrounds, we determined the nuclear genetic fingerprint of 16 short tandem repeats (STRs) (see Table S1). Adenocarcinoma A549 cybrids did not differ in their STR markers and did not vary from the 9 STR markers characterized from the ATCC adenocarcinoma A549 cell line. The same was true for the osteosarcoma 143B cybrids and the ATCC osteosarcoma 143B cell line.

Next, we confirmed the mtDNA alleles of the cybrids using PCR-RFLP. The m.8993T>G mutation caused the amplicon to be cut into two fragments, and the wild-type amplicon was not digested (see Figure S2). To rule out the presence of mtDNA nondefining haplogroup (private) mutations that can affect the bioenergetic phenotypes of these cybrids, we sequenced the complete mtDNA (GenBank JN635299, KJ742713, KJ742715, KT002148, KT002149) (see Table S2). Owt cybrids contained two nonsynonymous private mutations: m.3387T>A, provoking a p.MT-ND1:I27M substitution, and m.14189A>G, causing a p.MT-ND6:V162A change. Am cybrids contained one nonsynonymous private mutation: m.9194A>G, producing a p.MT-ATP6:H223R replacement. These mutations have previously been found zero, nine, and four times, respectively, in 29,867 mtDNA sequences (GenBank, February 2015). However, the affected positions show low evolutionary conservation (23.5%, 13.9%, and 14.1% in 5,165, 5,177, and 4,925 eukaryote species, respectively; GenBank, February 2015) when compared with the conservation of the p.MT-ATP6:L156 amino acid replacement (99.0% in 4,925 Eukaryota species) that causes MILS or NARP. This lack of evolutionary conservation suggests that these mutations would have little or no impact on phenotype. Therefore, we assumed that the m.8993T>G transversion was responsible for the major phenotypic differences between the mutant and wild-type cybrids that had the same nuclear background.

It has previously been reported that m.8993T>G mutant cybrids with the osteosarcoma 143B nuclear background showed reduced oxygen consumption (D’Aurelio et al. 2010; López-Gallardo et al. 2014; Pallotti et al. 2004), decreased ATP levels (D’Aurelio et al. 2010; Fujita et al. 2007; López-Gallardo et al. 2014; Pallotti et al. 2004; Sgarbi et al. 2009; Vazquez-Memije et al. 2009), diminished MIMP (López-Gallardo et al. 2014), and increased reactive oxygen species (ROS) (López-Gallardo et al. 2014; Wojewoda et al. 2010). Oxygen consumption was also reported to be reduced in m.8993T>G mutant cybrids with a cervical cancer HeLa EB8 nuclear background (Shidara et al. 2005). To confirm that the mutant cybrids used in the present study also showed reduced OXPHOS capability, we analyzed these mitochondrial variables. We found that oxygen consumption was significantly decreased in both Om and Am cybrids (see Figure S3A and Table S3). The ATP levels were significantly diminished only in the Om cybrids. In contrast with the Om cybrids, in which H_2_O_2_ levels were significantly higher than in Owti, H_2_O_2_ levels were decreased in Am cybrids compared with Awt, albeit not significantly so. It was previously reported that mitochondria in an m.8993T>G mutant osteosarcoma 143B cybrid generated MIMP at the same levels as the parental wild-type cells (Wojewoda et al. 2012). We found that, similar to the Om cybrids (López-Gallardo et al. 2014), the MIMP in Am cybrids was reduced (see Figure S3B), although this result was based on visual assessment of a single sample.

These results confirm that the biochemical phenotypes of our cybrids were similar to those of other reported cybrids with the m.8993T>G mutation.

### Effects of Tributyltin Chloride on Cybrids

**Figure 1:**
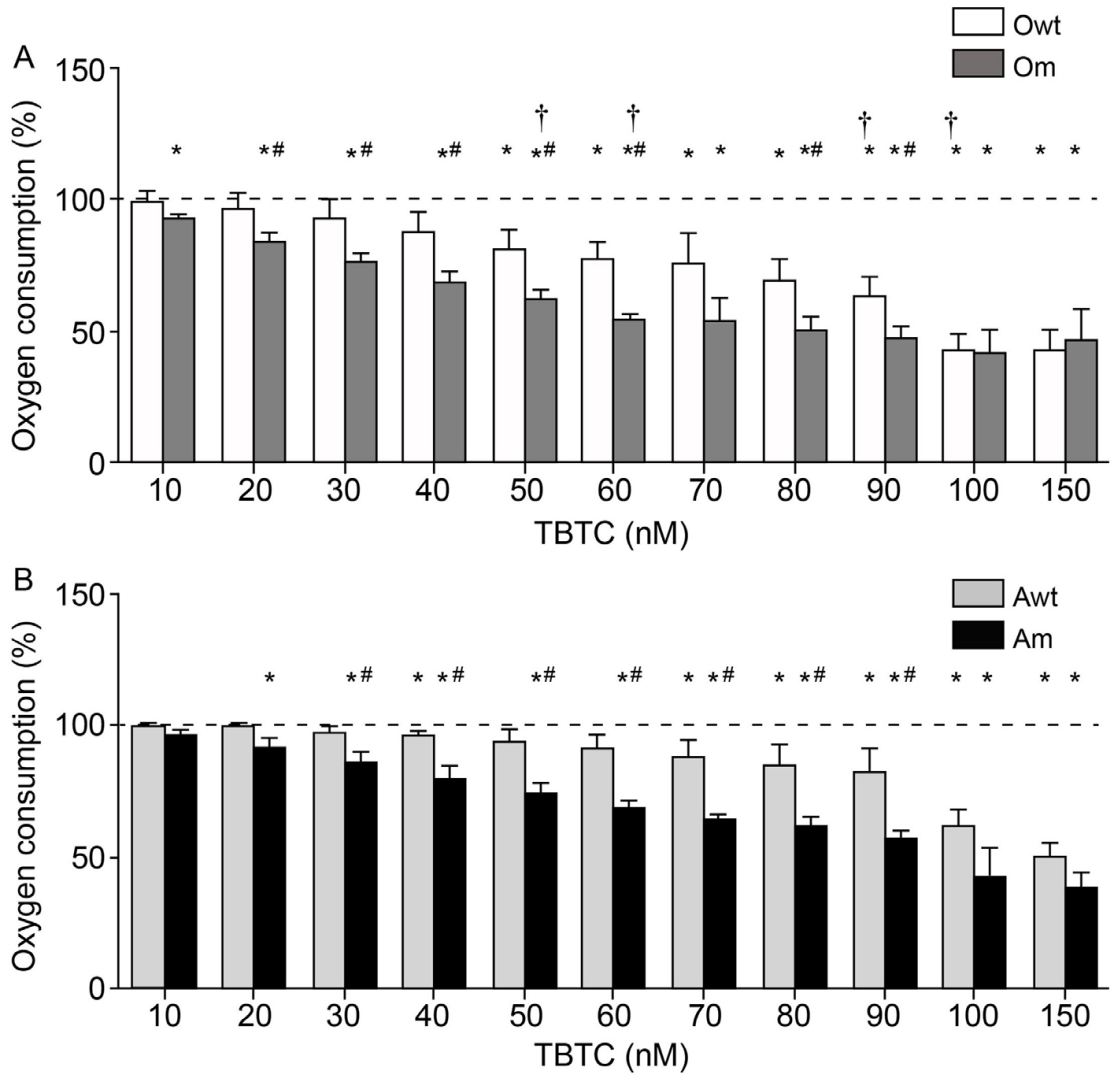
Oxygen consumption by osteosarcoma 143B (*A*) and adenocarcinoma A549 (*B*) cybrids. The dashed line (100%) represents the mean value in each of the untreated cybrids. The bars indicate the percentage of tributyltin chloride (TBTC)-treated cybrids. Error bars represent the standard deviation. Am, adenocarcinoma with mutation; Awt, wild-type adenocarcinoma; Om, osteosarcoma with mutation; Owt, nonisogenic wild-type osteosarcoma. *p < 0.05 versus the same untreated cybrid. ^#^ *p* < 0.05 versus the wild-type cybrid from the same nuclear background at the same TBTC concentration. ^†^ *p* < 0.05 for Om versus Am or Owt versus Awt at the same TBTC concentration.

It was previously reported that mitochondrial respiration was significantly decreased in mice treated with TBTC (Ueno et al. 2003) and that oxygen consumption in human adipose tissue–derived stem cells (hASCs) was decreased in reponse to 100 nM TBTC (Llobet et al. 2015b). We found that TBTC at concentrations ≥ 10 nM significantly decreased oxygen consumption in Om cybrids when compared with untreated cybrids (Figure 1A). TBTC ≥ 50 nM was required to significantly reduce oxygen consumption in Owt cybrids. There were negative and significant correlations between TBTC concentration and oxygen consumption in Owt [linear regression coefficient (β) for the change in %O_2_ consumption with a 1-nM increase in TBTC = –0.45; *R* ^2^ = 0.924; *p* < 0.0001] and Om (TBTC β = –0.41; *R* ^2^ = 0.796; *p* < 0.0001) cybrids. For the majority of TBTC concentrations between 20 and 90 nM, the reduction in oxygen consumption in response to TBTC was significantly greater in Om than in Owt cybrids.

Additionally, TBTC at concentrations ≥ 20 nM significantly decreased oxygen consumption in Am cybrids when compared with untreated cybrids (Figure 1B). TBTC ≥ 70 nM significantly reduced oxygen consumption in Awt cybrids; however, 40 nM TBTC also reduced this parameter. There were negative and significant correlations between TBTC concentration and oxygen consumption in Awt (TBTC β = –0.35; *R* ^2^ = 0.876; *p* < 0.0001) and Am cybrids (TBTC β = –0.46; *R* ^2^ = 0.956; *p* < 0.0001). For TBTC concentrations between 30 and 90 nM, the reduction in oxygen consumption in response to TBTC was significantly greater in Am than in Awt cybrids. The decrease in oxygen consumption in response to 50 and 60 nM TBTC was significantly greater in Om than in Am cybrids, and the decrease in oxygen consumption in response to 90 and 100 nM TBTC was significantly greater in Owt than in Awt cybrids.

**Figure 2:**
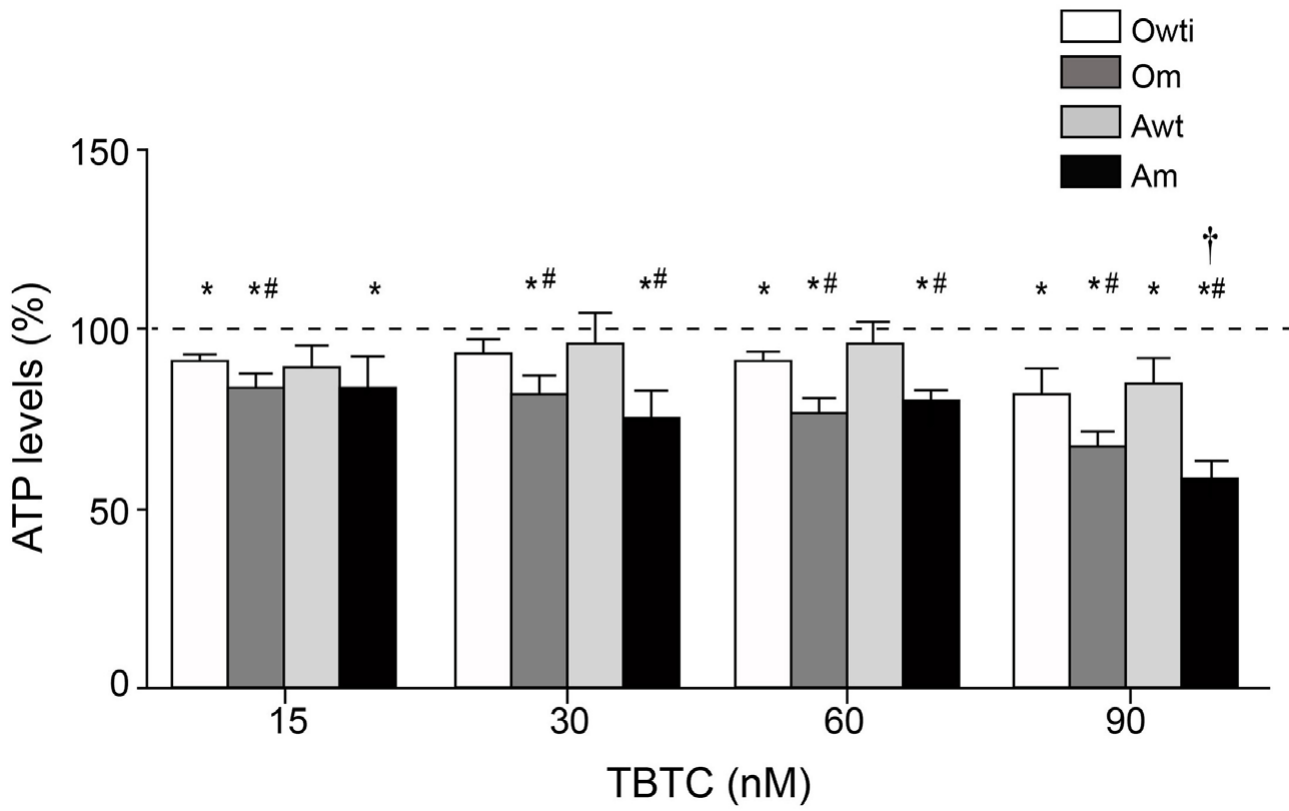
ATP levels in osteosarcoma 143B and adenocarcinoma A549 cybrids. The dashed line (100%) represents the mean value in each of the untreated cybrids. The bars indicate the percentage of tributyltin chloride (TBTC)-treated cybrids. Error bars represent the standard deviation. Am, adenocarcinoma with mutation; Awt, wild-type adenocarcinoma; Om, osteosarcoma with mutation; Owti, isogenic wild-type osteosarcoma. *p < 0.05 versus the same untreated cybrid. ^#^ *p* < 0.05 versus the wild-type cybrid from the same nuclear background at the same TBTC concentration. ^†^ *p* < 0.05 for Om versus Am at the same TBTC concentration.

Previous reports showed that 100 nM TBT decreased ATP levels in human embryonic carcinoma NT2/D1 cells (Yamada et al. 2014). We found that at concentrations ≥ 15 nM, TBTC significantly decreased ATP levels in Om and Am cybrids when compared with untreated cybrids (Figure 2). Furthermore, Owti and Awt cybrids treated with TBTC ≥ 60 nM and TBTC ≥ 90 nM, respectively, showed significantly reduced ATP levels. These levels were also reduced in Owti cybrids treated with 15 nM TBTC. There were negative and significant correlations between TBTC concentrations and ATP levels in Owti (TBTC β = –0.17; *R* ^2^ = 0.810; *p* = 0.0374), Om (TBTC β = –0.30; *R* ^2^ = 0.850; *p* = 0.0234), and Am cybrids (TBTC β = –0.38; *R* ^2^ = 0.780; *p* = 0.0491), but not in Awt cybrids (TBTC β = –0.11; *R* ^2^ = 0.382; *p* = 0.3076). Om and Am cybrids were more susceptible to TBTC concentrations ≥ 15 or ≥ 30 nM, respectively, than the wild-type cells. The decrease in ATP levels in response to 90 nM TBTC was significantly greater in Am than in Om cybrids. There were no significant differences in ATP levels between Owti and Awt cybrids at any TBTC dose.

**Figure 3:**
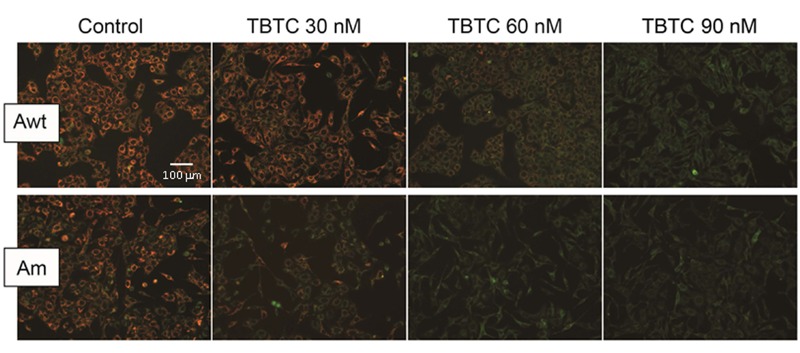
Mitochondrial inner membrane potential (MIMP) in single samples of tributyltin chloride (TBTC)-treated adenocarcinoma A549 cybrids. Reduced red staining corresponds to lower MIMP. Am, adenocarcinoma with mutation; Awt, wild-type adenocarcinoma.

In a previous study, MIMP was diminished in mouse thymocytes treated with 10 nM TBTC (Sharma and Kumar 2014). In the present study, visual assessment of a single sample suggested that at concentrations ≥ 30 nM, TBTC decreased the MIMP of the Am cybrid. However, a TBTC concentration ≥ 60 nM was required to affect the MIMP of the Awt cybrid (Figure 3).

**Figure 4:**
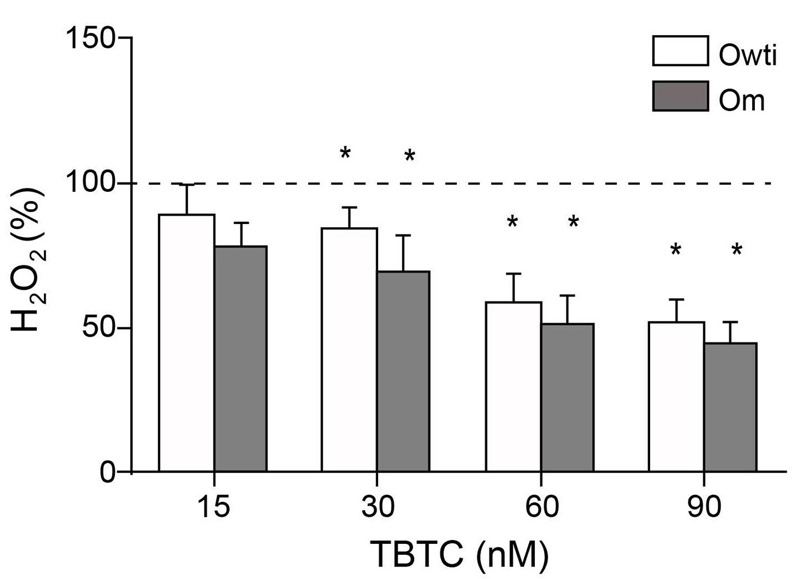
Hydrogen peroxide (H_2_O_2_) production in tributyltin chloride (TBTC)-treated osteosarcoma 143B cybrids. The dashed line (100%) represents the mean value in each of the untreated cybrids. The bars indicate the percentage of treated cybrids. Error bars represent the standard deviation. Om, osteosarcoma with mutation; Owti, isogenic wild-type osteosarcoma. *p < 0.05 versus the same untreated cybrid.

In a previous study, 10-nM concentrations of TBTC increased H_2_O_2_ production in mouse thymocytes (Sharma and Kumar 2014). However, in other studies, ≤ 300 nM TBTC did not increase H_2_O_2_ levels in a mixture of dissociated cells from different parts of rat brain (Mitra et al. 2014), and 100 nM TBTC did not change the H_2_O_2_ production in hASCs (Llobet et al. 2015b). We found that ≥ 30 nM TBTC significantly decreased H_2_O_2_ production in Owti and Om cybrids when compared with untreated cybrids (Figure 4). There were negative and significant correlations between TBTC concentrations and H_2_O_2_ levels in Owti (TBTC β = –0.56; *R* ^2^ = 0.969; *p* = 0.0006) and Om (TBTC β = –0.58; *R* ^2^ = 0.915; *p* = 0.0071) cybrids.

### Tributyltin Target

Several TBTC targets have been proposed. It has been reported that TBTC can activate genomic pathways by binding PPARγ (Kanayama et al. 2005). Thus, the different susceptibilities of mutant and wild-type cybrids to TBTC may be PPARγ-mediated. Osteosarcoma 143B and adenocarcinoma A549 cells express the PPARγ gene (Haydon et al. 2002; Li et al. 2014). However, we did not observe significant differences in PPARγ mRNA expression between mutant and wild-type cybrids (see Figure S3A). Therefore, this target does not explain our results.

**Figure 5:**
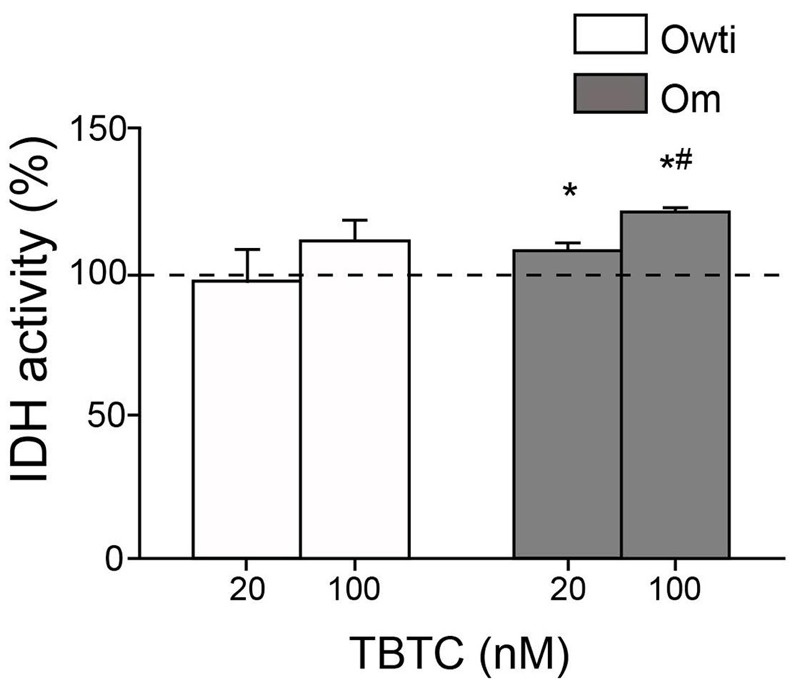
Isocitrate dehydrogenase (IDH) activity in tributyltin chloride (TBTC)-treated osteosarcoma 143B cybrids. The dashed line (100%) represents the mean value in each of the untreated osteosarcoma 143B cybrids. The bars indicate the percentage of treated cybrids. Error bars represent the standard deviation. Om, osteosarcoma with mutation; Owti, isogenic wild-type osteosarcoma. *p < 0.05 versus the same untreated cybrid. ^#^ *p* < 0.05 versus the mutant cybrid treated with 20 nM TBTC.

A previous report showed that 100 nM TBTC decreased IDH activity in human embryonic carcinoma NT2/D1 cells. These results suggest that IDH is a novel target of TBTC (Yamada et al. 2014). IDH is a mitochondrial enzyme from the tricarboxylic acid cycle that produces NADH. This compound is reoxidized in the OXPHOS electron transport chain and increases oxygen consumption and MIMP and ATP production. Therefore, inhibition of IDH by TBTC would decrease all of these parameters. However, we previously found that 100 nM TBTC increased IDH activity in hASCs (Llobet et al. 2015b). To assess IDH inhibition, we tested the effects of TBTC on osteosarcoma 143B cybrids. At TBTC concentrations ≥ 20 nM, Owti IDH activity did not differ significantly from that of untreated Owti cells. At concentrations of 20 and 100 nM, TBTC significantly increased IDH activity in the Om cybrid compared with untreated Om cells (Figure 5). These data suggest that NADH accumulation caused by OXPHOS dysfunction in mutant cybrids can provoke an abnormal increase in isocitrate and a compensatory expression of IDH. Indeed, the mutant cybrid showed significantly higher IDH activity than the Owti cybrid (see Figure S3A). Thus, inhibition of IDH does not explain our results.

**Figure 6:**
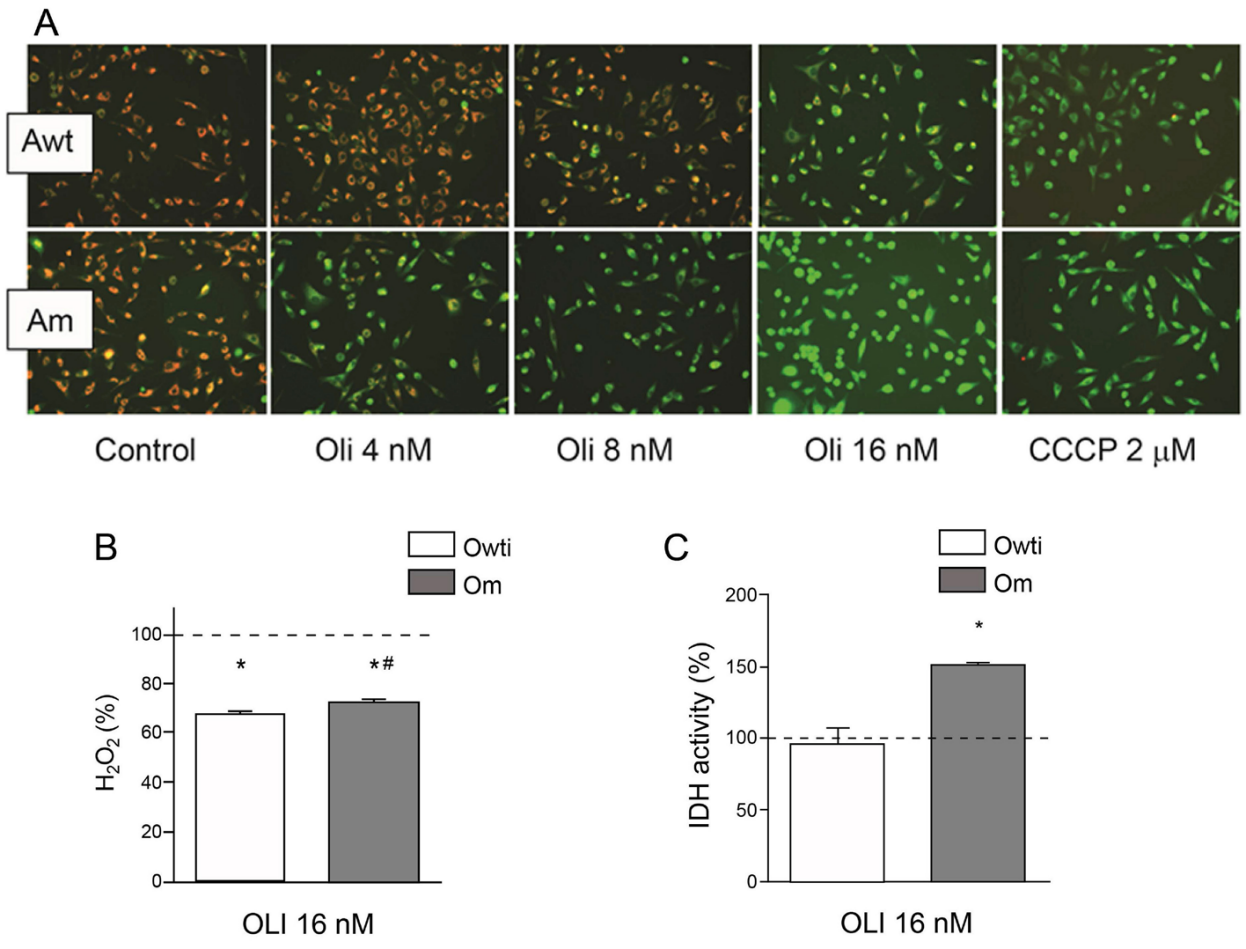
Effects of oligomycin (Oli) on different mitochondrial parameters. (*A*) Mitochondrial inner membrane potential (MIMP) in single samples of treated adenocarcinoma A549 cybrids. Reduced red staining corresponds to a lower MIMP. The uncoupler carbonyl cyanide *m*-chlorophenyl hydrazone (CCCP) was used as a control because it decreases MIMP. (*B*) Hydrogen peroxide (H_2_O_2_) production in treated osteosarcoma 143B cybrids. (*C*) Isocitrate dehydrogenase (IDH) activity in treated osteosarcoma 143B cybrids. The dashed line (100%) represents the mean value in each of the untreated osteosarcoma 143B cybrids. The bars indicate the percentage of treated cybrids. Error bars represent the standard deviation. Am, adenocarcinoma with mutation; Awt, wild-type adenocarcinoma; Om, osteosarcoma with mutation; Owti, isogenic wild-type osteosarcoma. *p < 0.05 versus the same untreated cybrid. ^#^ *p* < 0.05 versus the wild-type cybrid at the same OLI concentration.

CV is the third proposed target of TBTC. In previous studies, it was shown that, similarly to TBTC, at concentrations ≥ 49 nM, the CV inhibitor OLI decreased oxygen consumption in osteosarcoma 143B cybrids (Gómez-Durán et al. 2010, 2012; McKenzie et al. 2007; Zhang et al. 2012), and 3.15 μM OLI produced a decline in ATP production in these 143B cybrids (Gómez-Durán et al. 2010, 2012; McKenzie et al. 2007). Furthermore, 3.15 μM OLI increased and 6 μM OLI decreased MIMP in osteosarcoma 143B cybrids (McKenzie et al. 2007; Porcelli et al. 2009). Surprisingly, in the present study, a qualitative assessment suggested that MIMP was reduced in Awt cybrids in response to 16 nM OLI but was reduced in Am cybrids in response to only 4 nM OLI (Figure 6A). OLI may have caused a decrease in MIMP because there was insufficient ATP to activate respiratory substrates (Brown et al. 1990). The effect of OLI on H_2_O_2_ levels also varies. Previous studies showed that 4 nM OLI increased H_2_O_2_ levels in mouse preadipocytes 3T3-L1 (Carrière et al. 2003), and 16 nM OLI decreased H_2_O_2_ in hASCs (Llobet et al. 2015b). In both Om and Owt cybrids, 16 nM OLI decreased H_2_O_2_ levels (Figure 6B). Similar to its effect on hASCs (Llobet et al. 2015b), 16 nM OLI increased IDH activity in Om cybrids (Figure 6C). All of these results support CV as the target of TBTC (von Ballmoos et al. 2004). Further investigation is required to confirm the target of TBTC.

## Discussion

TBTC is a potent algicide and molluscicide that was used in marine antifouling ship paints (Grün 2014). Because of its adsorbing efficacy on sediments, long half-life, and lipophilic nature, levels of TBTC were considerably high in marine sediments and fishes (Mitra et al. 2014). Widespread environmental contamination of marine ecosystems with organotins began in the 1960s (Grün 2014). As a result, a global ban on the use of organotin-based antifouling paints was instituted from 2003 onward (Grün 2014). However, environmental contamination by organotins goes beyond aquatic ecosystems because these compounds are also used in industrial and agricultural activities (Grün 2014). Their slow rate of environmental degradation gives TBTC compounds the potential for bioaccumulation in upper trophic species of the food chain. Indeed, several studies have found TBT concentrations in human blood in the range of 8.3–293 nM (Kannan et al. 1999; Whalen et al. 1999). This range is consistent with the TBTC concentrations used in the present study. However, TBT compounds may be metabolized to dibutyltin and other metabolites (Boyer 1989), and TBT is rapidly cleared from blood. Thus, blood would not be the ideal biological compartment for estimating the butyltin burden in humans (Kannan et al., 1999). In liver samples from 9 Polish, 4 Japanese, and 18 Danish individuals, ranges of 2.4–11, 59–96, and 1.1–33 ng of butyltin (TBT + DBT + MBT)/g of liver were found (Kannan and Falandysz 1997; Nielsen and Strand 2002; Takahashi et al. 1999).

Om and Owt cybrids (or Am and Awt cybrids) differ in their mtDNA genotypes. The phenotypic differences provoked by TBTC between cybrids from the same nuclear genetic background suggest that mtDNA is the responsible factor. For the analysis of all mitochondrial variables, except oxygen consumption, in osteosarcoma 143B cybrids, we used isogenic cybrids (Owti and Om). The Owti cybrid differred from the Om cybrid only at the m.8993 nucleotide position. Our results suggest that this mtDNA nucleotide position is the factor responsible for the phenotypic differences in response to TBTC between these cells. Moreover, there was greater similarity in the mitochondrial response to TBTC between the Om and Am cybrids (or between Owt/Owti and Awt cybrids), which harbor different nDNA and mtDNA but the same m.8993 nucleotide, than between Om and Owt/Owti cybrids (or Am and Awt cybrids), which harbor the same nDNA or even the same mtDNA but differ at the m.8993 nucleotide. These results confirm that the m.8993T>G transversion was the responsible factor for the phenotypic differences between these cells in response to TBTC. Therefore, a combination of this pathological mutation and this environmental contaminant could modify the phenotypic expression of the m.8993T>G mutation.

Several p.MT-ATP6 TMH4 and 5 amino acids from the CV proton channel are mutated in patients suffering from different mitochondriopathies (López-Gallardo et al. 2014). The interaction of a particular mutation with an environmental factor, such as TBTC, may explain different pathological phenotypes. Interestingly, such an interaction might also explain why the m.9025G>A/p.MT-ATP6:G167S mutation, a candidate for the etiologic factor of a particular mitochondriopathy, is found in both patients and healthy individuals. The m.9025G>A mutation was found in a patient with loss of Purkinje cells (López-Gallardo et al. 2014), and Purkinje cells showed degenerative changes throughout tributyltin-treated rat cerebellum (Elsabbagh et al. 2002).

Organotin compounds are highly lipophilic and readily penetrate the blood-brain barrier to enter the brain (Kotake 2012). An *in vitro* study reported that at a concentration of 30 nM, TBTC caused a significant decrease in MIMP in dissociated mixed cells from different parts of the rat brain. Cells from the striatum showed a higher susceptibility than cells from other brain regions (Mitra et al. 2014). Brain lesions, particularly in the striatum, characterize a group of disorders termed “striatal necrosis syndromes.” These disorders include familial bilateral striatal necrosis (FBSN), MILS, and NARP, and they can be caused by *MT-ATP6* mutations (Schon et al. 2001). Interestingly, we found that levels of oxygen consumption, MIMP, and ATP production in wild-type cybrids exposed to TBTC were similar to levels in cybrids with *MT-ATP6* mutations before exposure to TBTC. More experiments in primary cells, such as neurons, are required. However, our findings suggest that environmentally induced striatal necrosis syndromes caused by xenobiotics, such as organotins, may be possible.

## Conclusion

Unusual exposures to environmental toxins, at appropriate times and concentrations, should also be considered in the diagnosis of rare diseases.

## Supplemental Material

(523 KB) PDF

## References

[r1] Boyer IJ (1989). Toxicity of dibutyltin, tributyltin and other organotin compounds to humans and to experimental animals.. Toxicology.

[r2] Brown GC, Lakin-Thomas PL, Brand MD (1990). Control of respiration and oxidative phosphorylation in isolated rat liver cells.. Eur J Biochem.

[r3] Carelli V Franceschini F Venturi S Barboni P Savini G Barbieri G 2007 Grand rounds: could occupational exposure to *n*-hexane and other solvents precipitate visual failure in Leber hereditary optic neuropathy? Environ Health Perspect 115 113 115, doi:10.1289/ehp.9245 17366829 PMC1797843

[r4] Carrière A, Fernandez Y, Rigoulet M, Pénicaud L, Casteilla L (2003). Inhibition of preadipocyte proliferation by mitochondrial reactive oxygen species.. FEBS Lett.

[r5] Chomyn A, Lai ST, Shakeley R, Bresolin N, Scarlato G, Attardi G (1994). Platelet-mediated transformation of mtDNA-less human cells: analysis of phenotypic variability among clones from normal individuals—and complementation behavior of the tRNA^Lys^ mutation causing myoclonic epilepsy and ragged red fibers.. Am J Hum Genet.

[r6] Cooper DN, Krawczak M, Polychronakos C, Tyler-Smith C, Kehrer-Sawatzki H (2013). Where genotype is not predictive of phenotype: towards an understanding of the molecular basis of reduced penetrance in human inherited disease.. Hum Genet.

[r7] D’Aurelio M, Vives-Bauza C, Davidson MM, Manfredi G (2010). Mitochondrial DNA background modifies the bioenergetics of NARP/MILS *ATP6* mutant cells.. Hum Mol Genet.

[r8] Degoul F, Diry M, Rodriguez D, Robain O, Francois D, Ponsot G (1995). Clinical, biochemical, and molecular analysis of a maternally inherited case of Leigh syndrome (MILS) associated with the mtDNA T8993G point mutation.. J Inherit Metab Dis.

[r9] Elsabbagh HS, Moussa SZ, El-tawil OS (2002). Neurotoxicologic sequelae of tributyltin intoxication in rats.. Pharmacol Res.

[r10] Enns GM, Bai RK, Beck AE, Wong LJ (2006). Molecular-clinical correlations in a family with variable tissue mitochondrial DNA T8993G mutant load.. Mol Genet Metab.

[r11] Fujita Y, Ito M, Nozawa Y, Yoneda M, Oshida Y, Tanaka M (2007). CHOP (C/EBP homologous protein) and ASNS (asparagine synthetase) induction in cybrid cells harboring MELAS and NARP mitochondrial DNA mutations.. Mitochondrion.

[r12] Gómez-Durán A, Pacheu-Grau D, López-Gallardo E, Díez-Sánchez C, Montoya J, López-Pérez MJ (2010). Unmasking the causes of multifactorial disorders: OXPHOS differences between mitochondrial haplogroups.. Hum Mol Genet.

[r13] Gómez-Durán A, Pacheu-Grau D, Martínez-Romero I, López-Gallardo E, López-Pérez MJ, Montoya J (2012). Oxidative phosphorylation differences between mitochondrial DNA haplogroups modify the risk of Leber’s hereditary optic neuropathy.. Biochim Biophys Acta.

[r14] Grün F (2014). The obesogen tributyltin.. Vitam Horm.

[r15] Hao XD, Yang YL, Tang NL, Kong QP, Wu SF, Zhang YP (2013). Mitochondrial DNA haplogroup Y is associated to Leigh syndrome in Chinese population.. Gene.

[r16] Haydon RC, Zhou L, Feng T, Breyer B, Cheng H, Jiang W (2002). Nuclear receptor agonists as potential differentiation therapy agents for human osteosarcoma.. Clin Cancer Res.

[r17] Holt IJ, Harding AE, Petty RK, Morgan-Hughes JA (1990). A new mitochondrial disease associated with mitochondrial DNA heteroplasmy.. Am J Hum Genet.

[r18] Kannan K, Falandysz J (1997). Butyltin residues in sediment, fish, fish-eating birds, harbour porpoise and human tissues from the Polish coast of the Baltic Sea.. Mar Pollut Bull.

[r19] Kannan K, Senthilkumar K, Giesy JP (1999). Occurrence of butyltin compounds in human blood.. Environ Sci Technol.

[r20] Kanayama T, Kobayashi N, Mamiya S, Nakanishi T, Nishikawa J (2005). Organotin compounds promote adipocyte differentiation as agonists of the peroxisome proliferator-activated receptor γ/retinoid X receptor pathway.. Mol Pharmacol.

[r21] Kotake Y (2012). Molecular mechanisms of environmental organotin toxicity in mammals.. Biol Pharm Bull.

[r22] Lake NJ, Bird MJ, Isohanni P, Paetau A (2015). Leigh syndrome: neuropathology and pathogenesis.. J Neuropathol Exp Neurol.

[r23] Li J, Chen L, Yu P, Liu B, Zhu J, Yang Y (2014). Telmisartan exerts anti-tumor effects by activating peroxisome proliferator-activated receptor-γ in human lung adenocarcinoma A549 cells.. Molecules.

[r24] Llobet L, Montoya J, López-Gallardo E, Ruiz-Pesini E (2015a). Side effects of culture media antibiotics on cell differentiation.. Tissue Eng Part C Methods.

[r25] Llobet L, Toivonen JM, Montoya J, Ruiz-Pesini E, López-Gallardo E (2015b). Xenobiotics that affect oxidative phosphorylation alter differentiation of human adipose-derived stem cells at concentrations that are found in human blood.. Dis Model Mech.

[r26] López-Gallardo E, Emperador S, Solano A, Llobet L, Martín-Navarro A, López-Pérez MJ (2014). Expanding the clinical phenotypes of *MT-ATP6* mutations.. Hum Mol Genet.

[r27] McFarland R, Clark KM, Morris AA, Taylor RW, Macphail S, Lightowlers RN (2002). Multiple neonatal deaths due to a homoplasmic mitochondrial DNA mutation.. Nat Genet.

[r28] McKenzie M, Liolitsa D, Akinshina N, Campanella M, Sisodiya S, Hargreaves I (2007). Mitochondrial ND5 gene variation associated with encephalomyopathy and mitochondrial ATP consumption.. J Biol Chem.

[r29] Mitra S, Siddiqui WA, Khandelwal S (2014). Early cellular responses against tributyltin chloride exposure in primary cultures derived from various brain regions.. Environ Toxicol Pharmacol.

[r30] Mkaouar-Rebai E, Chaari W, Younes S, Bousoffara R, Sfar MT, Fakhfakh F (2009). Maternally inherited Leigh syndrome: T8993G mutation in a Tunisian family.. Pediatr Neurol.

[r31] Montoya J, López-Gallardo E, Díez-Sánchez C, López-Pérez MJ, Ruiz-Pesini E (2009). 20 years of human mtDNA pathologic point mutations: carefully reading the pathogenicity criteria.. Biochim Biophys Acta.

[r32] Nielsen JB, Strand J (2002). Butyltin compounds in human liver.. Environ Res.

[r33] Pallotti F, Baracca A, Hernandez-Rosa E, Walker WF, Solaini G, Lenaz G (2004). Biochemical analysis of respiratory function in cybrid cell lines harbouring mitochondrial DNA mutations.. Biochem J.

[r34] Porcelli AM, Angelin A, Ghelli A, Mariani E, Martinuzzi A, Carelli V (2009). Respiratory complex I dysfunction due to mitochondrial DNA mutations shifts the voltage threshold for opening of the permeability transition pore toward resting levels.. J Biol Chem.

[r35] Prezant TR, Agapian JV, Bohlman MC, Bu X, Oztas S, Qiu WQ (1993). Mitochondrial ribosomal RNA mutation associated with both antibiotic-induced and non-syndromic deafness.. Nat Genet.

[r36] Rorbach J, Yusoff AA, Tuppen H, Abg-Kamaludin DP, Chrzanowska-Lightowlers ZM, Taylor RW (2008). Overexpression of human mitochondrial valyl tRNA synthetase can partially restore levels of cognate mt-tRNA^Val^ carrying the pathogenic C25U mutation.. Nucleic Acids Res.

[r37] Ruhoy IS, Saneto RP (2014). The genetics of Leigh syndrome and its implications for clinical practice and risk management.. Appl Clin Genet.

[r38] Schon EA, Santra S, Pallotti F, Girvin ME (2001). Pathogenesis of primary defects in mitochondrial ATP synthesis.. Semin Cell Dev Biol.

[r39] Schwartzentruber J, Buhas D, Majewski J, Sasarman F, Papillon-Cavanagh S, Thiffault I (2014). Mutation in the nuclear-encoded mitochondrial isoleucyl-tRNA synthetase *IARS2* in patients with cataracts, growth hormone deficiency with short stature, partial sensorineural deafness, and peripheral neuropathy or with Leigh syndrome.. Hum Mutat.

[r40] Sgarbi G, Casalena GA, Baracca A, Lenaz G, DiMauro S, Solaini G (2009). Human NARP mitochondrial mutation metabolism corrected with α-ketoglutarate/aspartate: a potential new therapy.. Arch Neurol.

[r41] Sharma N, Kumar A (2014). Mechanism of immunotoxicological effects of tributyltin chloride on murine thymocytes.. Cell Biol Toxicol.

[r42] Shidara Y, Yamagata K, Kanamori T, Nakano K, Kwong JQ, Manfredi G (2005). Positive contribution of pathogenic mutations in the mitochondrial genome to the promotion of cancer by prevention from apoptosis.. Cancer Res.

[r43] Takahashi S, Mukai H, Tanabe S, Sakayama K, Miyazaki T, Masuno H (1999). Butyltin residues in livers of humans and wild terrestrial mammals and in plastic products.. Environ Pollut.

[r44] Tatuch Y, Christodoulou J, Feigenbaum A, Clarke JT, Wherret J, Smith C (1992). Heteroplasmic mtDNA mutation (T----G) at 8993 can cause Leigh disease when the percentage of abnormal mtDNA is high.. Am J Hum Genet.

[r45] Thorburn DR, Rahman S (2014). Mitochondrial DNA-associated Leigh syndrome and NARP. In: *GeneReviews^®^ [Internet].* Pagon RA, Adam MP, Ardinger HH, Wallace SE, Amemiya A, Bean LJH, et al., eds.. http://www.ncbi.nlm.nih.gov/books/NBK1116/.

[r46] Tsao CY, Mendell JR, Bartholomew D (2001). High mitochondrial DNA T8993G mutation (<90%) without typical features of Leigh’s and NARP syndromes.. J Child Neurol.

[r47] Ueno S, Kashimoto T, Susa N, Shiota Y, Okuda M, Mutoh K (2003). Effects of butyltin compounds on mitochondrial respiration and its relation to hepatotoxicity in mice and Guinea pigs.. Toxicol Sci.

[r48] van Oven M, Kayser M (2009). Updated comprehensive phylogenetic tree of global human mitochondrial DNA variation.. Hum Mutat.

[r49] Vazquez-Memije ME, Rizza T, Meschini MC, Nesti C, Santorelli FM, Carrozzo R (2009). Cellular and functional analysis of four mutations located in the mitochondrial *ATPase6* gene.. J Cell Biochem.

[r50] von Ballmoos C, Brunner J, Dimroth P (2004). The ion channel of F-ATP synthase is the target of toxic organotin compounds.. Proc Natl Acad Sci USA.

[r51] Whalen MM, Loganathan BG, Kannan K (1999). Immunotoxicity of environmentally relevant concentrations of butyltins on human natural killer cells *in vitro*.. Environ Res.

[r52] Wojewoda M, Duszyński J, Szczepanowska J (2010). Antioxidant defence systems and generation of reactive oxygen species in osteosarcoma cells with defective mitochondria: effect of selenium.. Biochim Biophys Acta.

[r53] Wojewoda M, Duszyński J, Więckowski M, Szczepanowska J (2012). Effect of selenite on basic mitochondrial function in human osteosarcoma cells with chronic mitochondrial stress.. Mitochondrion.

[r54] Yamada S Kotake Y Demizu Y Kurihara M Sekino Y Kanda Y 2014 NAD-dependent isocitrate dehydrogenase as a novel target of tributyltin in human embryonic carcinoma cells. Sci Rep 4 5952, doi:10.1038/srep05952 25092173 PMC4121607

[r55] Zhang C, Huang VH, Simon M, Sharma LK, Fan W, Haas R (2012). Heteroplasmic mutations of the mitochondrial genome cause paradoxical effects on mitochondrial functions.. FASEB J.

