# Supplementary material for: Effects of Tributyltin Chloride on Cybrids with or without an ATP Synthase Pathologic Mutation
Source: Environ Health Perspect. 2016 Apr 29;124(9):1399–405. doi: 10.1289/EHP182 (PMC5010394; doi:10.1289/EHP182)
Supplement: (523 KB) PDF [file EHP182.s001.acco.pdf]

Environ Health Perspect
DOI: 10.1289/EHP182

Note to readers with disabilities: *EHP* strives to ensure that all journal content is accessible to all readers. However, some figures and Supplemental Material published in *EHP* articles may not conform to 508 standards due to the complexity of the information being presented. If you need assistance accessing journal content, please contact ehp508@niehs.nih.gov. Our staff will work with you to assess and meet your accessibility needs within 3 working days.

Supplemental Material

Effects of Tributyltin Chloride on Cybrids with or without an ATP Synthase Pathologic Mutation

Ester López-Gallardo, Laura Llobet, Sonia Emperador, Julio Montoya, and Eduardo Ruiz-Pesini

Table of Contents

Figure S1. Characterization of the cybrids' nuclear genetics backgrounds. Representative images of metaphase preparations (karyotypes) from different cybrids. Chromosome modal number and range (in brackets) are indicated.

Figure S2. Characterization of the cybrids' mitochondrial genetics backgrounds. Analysis of the m.8993 genotype. m.8993T and m.8993G amplicons generate one or two fragments, respectively, after digestion with HpaII. MWM codes for molecular weight marker.

Figure S3. Bioenergetic parameters in mutant and wild-type cybrids. A) Oxygen consumption, ATP amount, MIMP, H_2O_2 levels, PPARγ mRNA quantity and IDH activity. The dashed line (100 %) represents the mean values of these variables in each of the wild-type cybrids. The bars indicate the percentage of mutant cybrids. Error bars represent the standard deviation. In the determination of H_2O_2 production, the fluorescence intensity of 2',7'-Dichlorofluorescein in adenocarcinoma A549 cybrids is 100 times lower than that in osteosarcoma 143B cybrids. This is probably responsible for the large standard deviations observed. *, $P < 0.05$ vs. the wild-type cybrid from the same nuclear background. Original data of these cybrids can be obtained in Supplemental Material, Table S3. B) MIMP in single samples of adenocarcinoma A549 cybrids. Lesser red stain corresponds to lower MIMP.

DOI: 10.1289/EHP182

Table S1. Characterization of the cybrids' nuclear genetics backgrounds. Genetic fingerprints.
Table S2. Characterization of the cybrids' mitochondrial genetics backgrounds (mtDNA haplotypes).
Table S3. Numeric data for wild-type osteosarcoma 143B and adenocarcinoma A549 cybrids shown in Figure S3A.

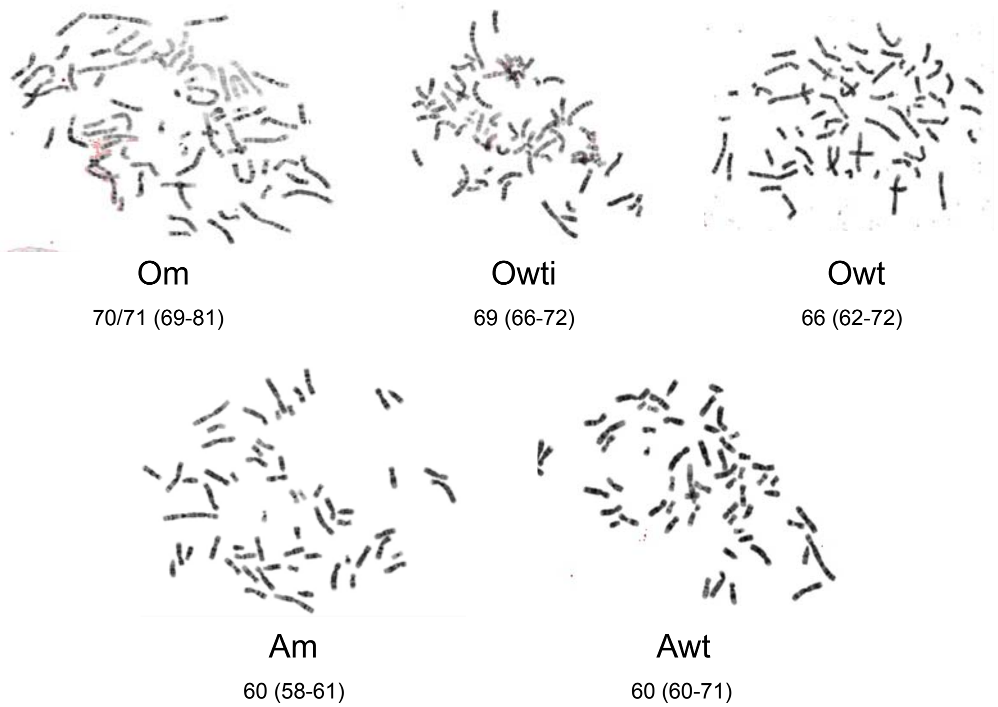

Figure S1. Characterization of the cybrids' nuclear genetics backgrounds. Representative images of metaphase preparations (karyotypes) from different cybrids. Chromosome modal number and range (in brackets) are indicated.

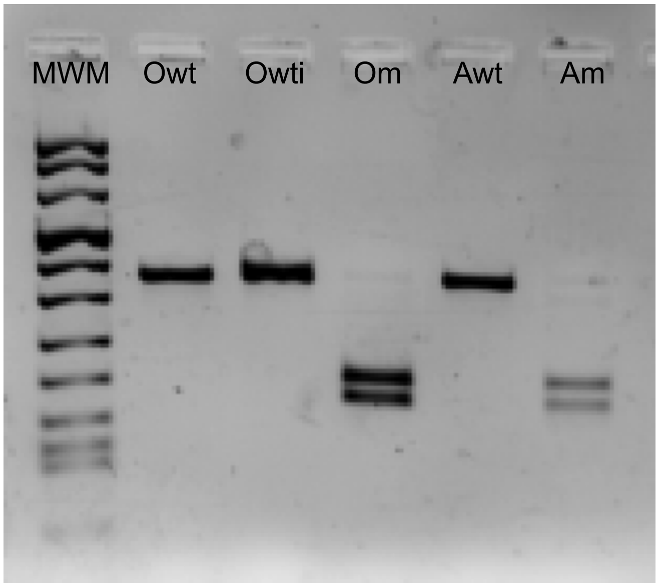

Figure S2. Characterization of the cybrids' mitochondrial genetics backgrounds. Analysis of the m.8993 genotype. m.8993T and m.8993G amplicons generate one or two fragments, respectively, after digestion with HpaII. MWM codes for molecular weight marker.

A

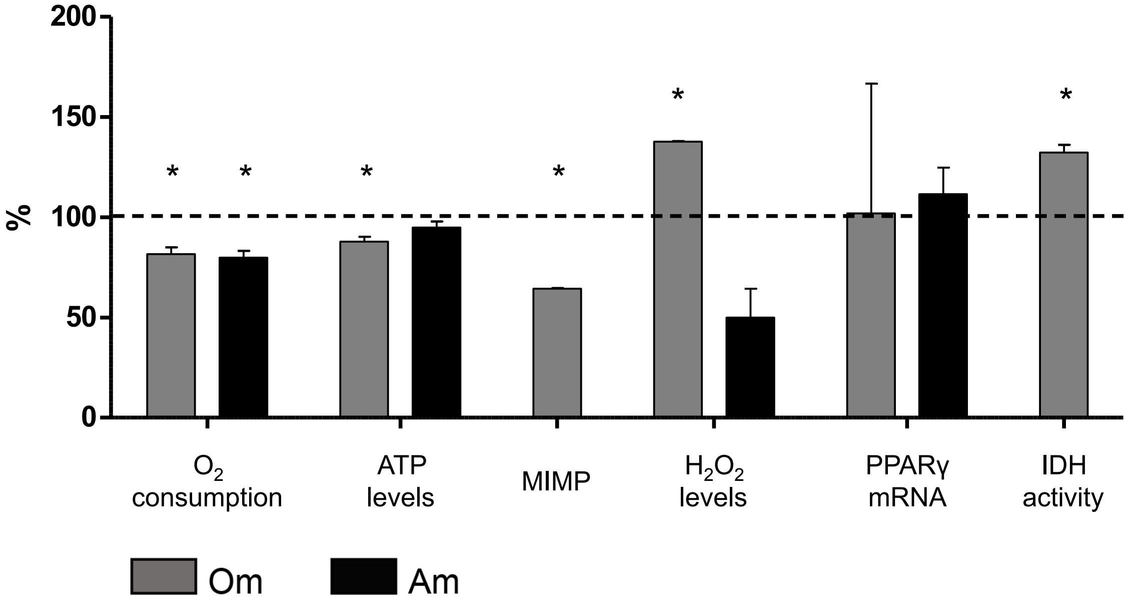

B

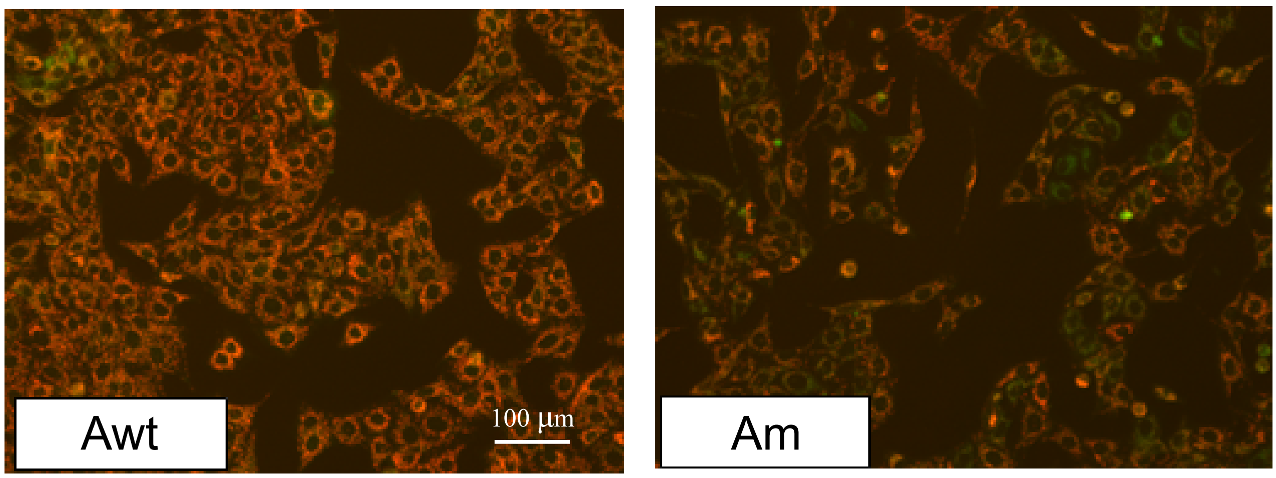

Figure S3. Bioenergetic parameters in mutant and wild-type cybrids. A) Oxygen consumption, ATP amount, MIMP, H_2O_2 levels, PPARγ mRNA quantity and IDH activity. The dashed line (100 %) represents the mean values of these variables in each of the wild-type cybrids. The bars indicate the percentage of mutant cybrids. Error bars represent the standard deviation. In the determination of H_2O_2 production, the fluorescence intensity of 2',7'-Dichlorofluorescein in adenocarcinoma A549 cybrids is 100 times lower than that in osteosarcoma 143B cybrids. This is probably responsible for the large standard deviations observed. *, $P < 0.05$ vs. the wild-type cybrid from the

DOI: 10.1289/EHP182

same nuclear background. Original data of these cybrids can be obtained in Supplemental Material, Table S3. B) MIMP in single samples of adenocarcinoma A549 cybrids. Lesser red stain corresponds to lower MIMP.

Environ Health Perspect
DOI: 10.1289/EHP182

Table S1. Characterization of the cybrids' nuclear genetics backgrounds. Genetic fingerprints.

Chromosome	Marker	Osteosarcoma 143B				Adenocarcinoma A549		
		Om	Owti	Owt	ATCC	Am	Awt	ATCC
2	D2S1338	24,25	24,25	24,25		24,25	24,25	
2	TPOX	11	11	11	11	8,11	8,11	8,11
3	D3S1358	15	15	15		16	16	
4	FGA	24	24	24		23	23	
5	D5S818	13	13	13	13	11	11	11
5	CSF1PO	12	12	12	12	10,12	10,12	10,12
7	D7S820	11,12	11,12	11,12	11,12	8,11	8,11	8,11
8	D8S1179	11,14	11,14	11,14		13,14	13,14	
11	TH01	6	6	6	6	8,9.3	8,9.3	8,9.3
12	vWA	18	18	18	18	14	14	14
13	D13S317	12	12	12	12	11	11	11
16	D16S539	10,13	10,13	10,13	10,13	11,12	11,12	11,12
18	D18S51	17	17	17		14,17	14,17	
19	D19S433	13	13	13		13,14	13,14	
21	D21S11	31.2,32.2	31.2,32.2	31.2,32.2		29	29	
X	AMEL	X	X	X	X	X,Y	X,Y	X,Y

Numbers in the body of the table represent short tandem repeats (STR) alleles.
Om, Owti, Owt, Am, and Awt code for mutant osteosarcoma 143B, isogenic wild-type osteosarcoma 143B, wild-type osteosarcoma 143B, mutant adenocarcinoma A549 and wild-type adenocarcinoma A549 cybrids, respectively.
ATCC represents the genetic fingerprints for osteosarcoma 143B and adenocarcinoma A549 cell lines reported by the American Tissue Culture Collection.

Table S2. Characterization of the cybrids' mitochondrial genetics backgrounds (mtDNA haplotypes).

mtDNA nucleotide	**rCRS** (NC012920)	**Owt** (JN635299)	**Owti** (KJ742715/ JX082001)	**Om** (KJ742713)	**Awt** (KT002149)	**Am** (KT002148)
73	A	G			G	
185	G	A				
228	G	A				
263	A	G	G	G	G	G
295	C	T				
309-310	CT	CCT	CCCT	CCCT	CCT	
315-316	CG	CCG	CCG	CCG	CCG	CCG
462	C	T				
465	C		T	T		
489	T	C				
750	A	G	G	G	G	G
1438	A	G	G	G	G	G
1700	T				C	
2392	T					C
2706	A	G			G	
3010	G	A	A	A		
3197	T				C	
3387	T	A				
4216	T	C				
4386	T					C
4769	A	G	G	G	G	G
6776	T					C
7028	C	T			T	
8723	G		A	A		
8860	A	G	G	G	G	G
8993	T			G		G
9194	A					G
9477	G				A	
10084	T	C				
10724	T					C
11251	A	G				
11467	A				G	
11719	G	A			A	
12308	A				G	
12346	C				T	

DOI: 10.1289/EHP182

12372	G				A	
12612	A	G				
12771	G		A	A		
13105	A				G	
13617	T				C	
13708	G	A				
14189	A	G				
14766	C	T			T	
14793	A				G	
14798	T	C				
15148	G				A	
15217	G		A	A		
15218	A				G	
15326	A	G	G	G	G	G
15452	C	A				
15530	T				C	
15553	G		A	A		
16069	C	T				
16126	T	C				
16241	A	G				
16256	C				T	
16270	C				T	
16274	G		A	A		
16319	G	A				
16399	A				G	
16519	T		C	C		C
Haplogroup	H2a2a1	J1c8a	H1ae	H1ae	U5a1a2b	H3ab

rCRS codes for revised Cambridge Reference Sequence.
Om, Owti, Owt, Am, and Awt code for mutant osteosarcoma 143B, isogenic wild-type osteosarcoma 143B, wild-type osteosarcoma 143B, mutant adenocarcinoma A549 and wild-type adenocarcinoma A549 cybrids, respectively.
GenBank accession numbers are provided in brackets.
Blank cells indicate wild-type allele (same than rCRS).

Table S3. Numeric data for wild-type osteosarcoma 143B and adenocarcinoma A549 cybrids shown in Figure S3A.

	**Owt**	**Owti**	**Awt**
O_2 consumption (pmol/s * 10^6 cells)	76 ± 13.3 (3)	--	88 ± 5.8 (3)
ATP levels (RLU)	--	15.01 ± 0.65 (3)	6.54 ± 0.32 (3)
MIMP (RFU)	--	18 ± 0.4 (3)	--
H_2O_2 (RFU)	--	22,818 ± 381 (3)	173.1 ± 70.8 (3)
PPARγ (ΔCt)	--	12.66 ± 0.57 (3)	13.05 ± 0.63 (3)
IDH (mOD/min * cell)	--	1.7 ± 0.19 (3)	--

Owt, Owti, and Awt code for wild-type osteosarcoma 143B, isogenic wild-type osteosarcoma 143B, and wild-type adenocarcinoma A549 cybrids, respectively.
RLU, RFU, ΔCt and mOD code for relative luminescence units, relative fluorescence units, change in cycle threshold and millioptic density.
As commented in the “Results” section, we first built a wild-type cybrid with the osteosarcoma 143B nuclear background (Owt) using platelets from a control individual. Then, we had the opportunity to obtain platelets from the healthy mother of the m.8993T>G patient. She has the same mitochondrial genotype but without the pathologic mutation. Therefore, we built a second wild-type cybrid with the osteosarcoma 143B nuclear background (Owti) more genetically similar to the mutant cybrid. This is the reason to use Owti for most of the comparisons, after using Owt for oxygen consumption that was performed before having the second cybrid.
As it is thought that MIMP of m.8993G cells should be higher than that of m.8993T cells, but we observed just the opposite in our Om and Owti cybrids, we wanted to show a visual result (although no quantitative). This is the reason why we only analyzed adenocarcinoma A549 cybrids in this way and do not show quantitative results in this table.
Contrary to the reported inhibitory effect of TBTC on IDH activity, and as already commented in the “Results” section, we had previously observed that TBTC increased IDH activity in human adipose-derived stem cells. We obtained the same result for Om cybrids and no change in Owt. Therefore, we considered that these results were enough to rule out IDH as the TBTC target, we do not repeat the analysis in adenocarcinoma A549 cybrids, and do not have results to show in this table.
Data for mean, standard deviation, and number of samples analyzed (in brackets) are presented.
